# Impact of Local and Demographic Factors on Early COVID-19 Vaccine Hesitancy among Health Care Workers in New York City Public Hospitals

**DOI:** 10.3390/vaccines10020273

**Published:** 2022-02-10

**Authors:** Afsheen Afzal, Masood A. Shariff, Victor Perez-Gutierrez, Amnah Khalid, Christina Pili, Anjana Pillai, Usha Venugopal, Moiz Kasubhai, Balavenkatesh Kanna, Brian D. Poole, Brett E. Pickett, David S. Redd, Vidya Menon

**Affiliations:** 1 Department of Medicine, NYC Health and Hospitals/Lincoln, Bronx, NY 10451, USA; afzala1@nychhc.org (A.A.); shariffm@nychhc.org (M.A.S.); perezgv@nychhc.org (V.P.-G.); khalida8@nychhc.org (A.K.); anjana.pillai@nychhc.org (A.P.); Usha.Venugopal@nychhc.org (U.V.); Moiz.Kasubhai@nychhc.org (M.K.); balavenkatesh.kanna@nychhc.org (B.K.); 2 Research Administration, NYC Health and Hospitals/Central Office, New York, NY 10013, USA; Christina.pili@nychhc.org; 3 Department of Microbiology and Molecular Biology, Brigham Young University, Provo, UT 84602, USA; brett_pickett@byu.edu (B.E.P.); dsr.1991@gmail.com (D.S.R.)

**Keywords:** COVID-19, vaccine attitudes, healthcare workers

## Abstract

Despite the development of several effective vaccines, SARS-CoV-2 continues to spread, causing serious illness among the unvaccinated. Healthcare professionals are trusted sources of information about vaccination, and therefore understanding the attitudes and beliefs of healthcare professionals regarding the vaccines is of utmost importance. We conducted a survey-based study to understand the factors affecting COVID-19 vaccine attitudes among health care professionals in NYC Health and Hospitals, at a time when the vaccine was new, and received 3759 responses. Machine learning and chi-square analyses were applied to determine the factors most predictive of vaccine hesitancy. Demographic factors, education, role at the hospital, perceptions of the pandemic itself, and location of work and residence were all found to significantly contribute to vaccine attitudes. Location of residence was examined for both borough and neighborhood, and was found to have a significant impact on vaccine receptivity. Interestingly, this borough-level data did not correspond to the number or severity of cases in the respective boroughs, indicating that local social or other influences likely have a substantial impact. Local and demographic factors should be strongly considered when preparing pro-vaccine messages or campaigns.

## 1. Introduction

New York City was the epicenter of the COVID-19 pandemic in the US in early 2020. As the primary provider of health services for the city’s vulnerable patient population, the city’s public health system (NYC Health and Hospitals) bore a disproportionate burden of the pandemic [1]. The healthcare workers of the system responded heroically to the unprecedented challenge to ensure the best care for their patients, in spite of facing potential exposure to the virus through their jobs. While there exists a significant gap in data collection, it has been shown that the majority of cases and deaths related to COVID-19 among healthcare workers (HCW) have been in people of color who work in areas where risk of workplace exposure is high [2].

Vaccines have historically had a significant positive impact on public health, reducing disease transmission and/or decreasing the incidence of serious diseases such as polio, smallpox, diphtheria, hepatitis, and others [1]. In December 2020, the U.S. Food and Drug Administration (FDA) issued Emergency Use Authorizations (EUA) for the first two COVID-19 vaccines [3]. The Pfizer-BioNTech COVID-19 vaccine received an EUA on December 11th and the Moderna vaccine received an EUA on December 18th [3]. The Advisory Committee on Immunization Practices (ACIP) developed a framework (for the CDC) to prioritize vaccination by dividing the population groups into phases. Healthcare personnel and residents of long-term care facilities were included in Phase 1a of the vaccination program [4]. Health + Hospitals implemented an extraordinary vaccination effort based on the guidance from CDC to ensure equitable distribution of the vaccines. In spite of the scientific evidence on the safety and efficacy of the vaccines and the risk of severe illness and death associated with COVID-19 infection, vaccine hesitancy/refusal was observed among the HCWs. 

Attitudes towards SARS-CoV2 vaccines among HCW has been extensively studied, with the timing of the study ranging from prior to COVID-19 vaccine roll out (mid to late 2020) to late 2021 [2,3,4]. Data of COVID-19 infection among HCW is not consistent, and the ECDC has reported a range HCW infection range from 2.2–29% [5]. In spite of the experiences of being at the front lines of the pandemic, striking heterogeneity towards vaccination has been observed among HCWs in studies from France, Italy and Canada [6,7,8]. In a survey-based study from Italy on the attitudes of HCW towards SARS-CoV2 vaccination, 67% intended to be vaccinated, while 26% were hesitant and 7% refused to be vaccinated [9]. The overall rate of hesitancy towards SARS-CoV2 vaccines among HCW in this study was comparable to that of the general population in Italy as described in two multinational surveys [10,11]. The rapid development of these vaccines and the fear of adverse effects were reported as the most common reasons for vaccine hesitancy among the HCW [9]. While imposition of vaccine mandates, green pass implementation, domestic vaccine passports etc. [12] has increased uptake of vaccination, there is increasing distrust among the people due to the reduction in individual autonomy resulting in polarization of the population nationally and internationally.

One area that is lacking, however, is an understanding of how regional effects can influence vaccination patterns [13], especially in health care workers. New York City is by no means uniform, with racial, ethnic, educational, socioeconomic, and other disparities present between various areas of the city. Even within the boroughs, the neighborhoods are often quite different. There is a consensus that vaccination is the best and fastest way out of the pandemic. NYC rolled out vaccination for HCW in mid-December 2020. The vaccination was rolled out in phases. Our study was done between February to March 2021. Though NYC and our health care system had borne the worst brunt of the pandemic, our initial observations had demonstrated significant COVID-19 vaccine hesitancy [2]. With this background we intended to explore the impact of the local/regional influences among our HCW affecting their decision to get vaccinated.

Our cross-sectional survey across all eleven acute care facilities of New York City Health + Hospital aimed to investigate attitudes towards vaccines among health care professionals. We hypothesized that exposure to the effects of COVID-19 would have a substantial impact on their willingness to be vaccinated [14]. We further hypothesized that where the respondents lived and worked in New York City would have a significant effect on vaccination status and attitudes. Understanding locational effects could guide policymakers and local public health officials in outreach efforts not only to area hospitals, but in understanding the individual concerns of specific neighborhoods or making sure that access to and information about vaccines is provided to these neighborhoods.

## 2. Materials and Methods

### 2.1. Settings and Survey Participants

Healthcare workers from all facilities of Health + Hospitals participated in a cross-sectional study by completing an online survey developed to understand their knowledge, attitude and perceptions about the COVID-19 vaccination. This survey was sent out via the intranet twice a week for one month. They were provided with an anonymous link to the survey, using the Survey Monkey platform. Only complete responses were recorded and counted. Surveys completed by respondents with the same IP address were excluded as they were considered as an overlapping response. No cap was set on participation. Selection bias was avoided by sending the survey to all employees of the health system. Institutional Review Board (IRB) approval was obtained (IRB protocol #20-043). 

### 2.2. Survey

The survey was constructed to assess the participants’ knowledge and attitudes about the impact of COVID-19 infection, vaccinations in general, and COVID-19 vaccination. It consisted of 23 questions and was administered from late February to late March 2021. The survey started with a question to determine if the respondent has received the COVID-19 vaccine. For those who answered “Yes”, the next question attempted to evaluate the reason(s) for getting the vaccine. For those who answered “No” to the first question, the follow-up question was if they will get the vaccine in the next two months. Respondents who did not agree to get vaccinated in the next two months were considered as “refuse to get vaccinated” while those who “did not know” about whether they wanted to get vaccinated in the next two months, were considered as “hesitant”. Reason(s) for their decision to not get vaccinated were sought from the respondents who refused to get the vaccine, while the hesitant group was asked about “what would help them make a decision about getting the vaccine”. Previously published data that personal exposure to individuals suffering from COVID-19 would decrease vaccine hesitancy, respondents were asked to identify the most severe outcome of infection among people they knew personally, as well as the closeness of the relationship [14]. Attitude towards vaccinations in general was also determined via the survey. Sociodemographic characteristics including age, gender, race, marital status, number of children, level of education, primary role, location of work, residential ZIP code and primary source of COVID-19-related information were obtained from all participants. The entire survey is available in the supplementary section (Table S1).

The survey was based on questions derived from a prior survey, which was validated via exploratory factor analysis and confirmatory factor analysis [2].

Health + Hospitals, the nation’s largest public health care delivery system is an integrated network of hospitals, trauma centers, neighborhood health centers, nursing homes, and post-acute care centers and employs a workforce of more than 35,000. To ensure that the survey was easily understood by the majority of the Health + Hospitals employees, the questions were framed for easy comprehension of participants with a minimum reading level of grade 4. Following the development of the questionnaire, experts from Popultation Health, Public Relations, Research, Human Resources reviewed and vetted the questions for content, lucidity and accuracy. It was then piloted on a small group of HCW including medical students, residents, administrative staff, clinical research team etc., and their feedback/suggestions were used to improve the final survey.

### 2.3. Random Forest Analysis

Machine learning was applied to a table that included the anonymized responses for all questions (columns) across all participants (rows). To evaluate responses to the statement “I will get the vaccine within the next two months”, all “Yes” or “NA” answers were removed so that only the “No” and “undecided” answers remained. Empty values in columns with incomplete responses were imputed using the average value across the column. This is required since random forest requires data to be present for all cells in the table. Random forest analysis (Random Forest version 4.6–14 library in R) was used to generate and analyze 10,000 trees with 10 variables tried at each split. The value imputation further reduced the noise since many columns only had a handful of responses, which were generally represented by the same answer. This effectively removed these columns from consideration. After this process, there were 91 columns that remained in the analysis, compared to the original 115 columns. The importance of each feature was ranked according to the mean decrease in Gini value, which was computed by the model generated by the random forest algorithm. These data were then used to generate a receiver-operator characteristic (ROC) curve to quantify the true-positive and false-positive rates of the model.

### 2.4. Statistical Analysis

The hospital system employed 35,000 employees and the sample size was calculated with a confidence level of 95% and a 2% margin of error to give a calculated sample size of 2247 participants. Chi-square (χ2) analysis was used to analyze dependency of variables between different groups when the variables were discreet. When the variables were continuous, ANOVA was used to determine whether any statistically significant differences were detectable. Neighborhoods within New York City where the respondents lived were identified using ZIP (Postal) codes [15]. The magnitude of the effect for each vaccine-hesitant group was calculated by modifying the method of Sullivan and Feinn [16]. The median of each group was calculated, then we used the formula $\frac{item-median}{interquartile\;range}$. This shows the proportion of the interquartile range from the median for each item, making effect size apparent and allowing a comparison across groups.

## 3. Results

### 3.1. Study Population

Of the 38,000 individuals who were sent a link to the survey, we received 3759 responses, a rate of approximately 10%. We therefore began by summarizing the wide range of racial/ethnic and educational backgrounds represented by the survey participants, as shown in Table 1.

This section may be divided by subheadings. It should provide a concise and precise description of the experimental results, their interpretation, as well as the experimental conclusions that can be drawn.

Since we sent the survey to the entire healthcare system, a wide variety of different roles within the hospital were included in the responses. To better understand the distribution of roles, we summarized the employment roles of respondents (Table 2). We observed that nurses were the largest group represented, with 17.6% of respondents, followed by physicians, with 17.1%. Administrative support staff were the next largest group of respondents, and many other roles were represented.

### 3.2. Vaccine Status

We administered this survey after preliminary approval of the COVID-19 vaccines, when the vaccines were available primarily to healthcare personnel. Of those who participated in the survey, 71% had already received their first vaccination dose. Of those who had not been vaccinated, 33% planned on being vaccinated, while 21.4% were not and 45.6% were unsure. For the purposes of this paper, “vaccinated”, “hesitant”, “hesitancy” or “refusal” refers only to the COVID-19 vaccine. When the respondents’ attitudes toward vaccines in general is relevant, it will be indicated. 

### 3.3. Demographics

Age, race, and gender were all significantly associated with vaccine hesitancy and vaccine refusal (*p* < 0.001 for all groups) (Table 3). The youngest age group (18–24) was the least likely to be vaccinated, and the most likely to be hesitant or refuse vaccination. The over-65 age group was the most likely to be vaccinated. Hesitancy among the other age ranges was not substantially different in magnitude. 

We discovered that race was also significantly associated with both vaccine hesitancy and refusal. Asian and White participants had the highest level of vaccination, with Black, Native American, and “Other” participants at the lower end. Gender was significantly associated with vaccination status, with men being more likely than women to be vaccinated (87% vs. 77%). Non-binary and “prefer not to answer” participants were the most likely to be unvaccinated, although their numbers were much smaller.

### 3.4. Education and Hospital Role

Level of education was significantly associated with vaccine hesitancy and refusal (*p*-value < 0.001). Generally, the more education a person had obtained, the more likely they were to be vaccinated, with the exception of those with “some high school” for which the numbers were too small to be meaningful. Interestingly, among those who were not vaccinated, respondents with a high school diploma or GED were more likely to be hesitant (24% for each) than refusers, while those with some college, an associate’s degree, or a bachelor’s degree all had higher rates of refusal than the high school or GED individuals, even though their overall vaccination rates were higher (Table 4). 

The respondent’s role in the hospital was significantly associated with the vaccination status of the person. Medical staff (including physicians) were highest at 95%, while the hospital police were the lowest, at 35% vaccinated. Clinical support staff were next highest, at 86% vaccinated. Nurses were 75% vaccinated, 16% hesitant, and 8% refusers. Interestingly, considering the nature of their jobs, community outreach tracers were only 64% vaccinated and 32% hesitant (Table 4).

### 3.5. Reasons for Hesitancy towards the COVID-19 Vaccine

**Figure 1:**
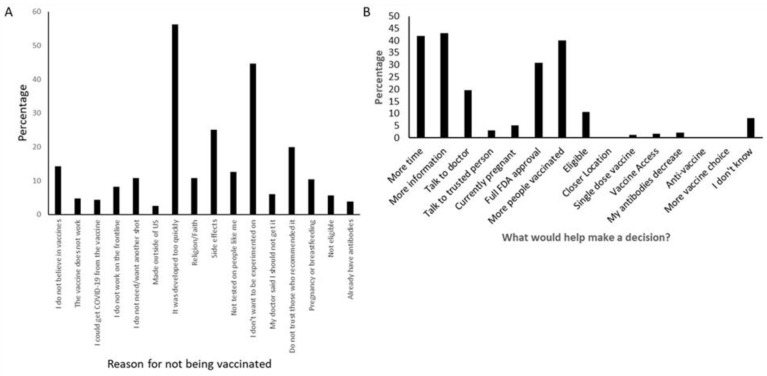
**Reasons for vaccine attitudes and behaviors**. (**A**). Unvaccinated respondents who indicated that they would not be vaccinated in the next three months (refusers) were asked why (*n* = 231). (**B**). Those HCW who did not receive the first dose of the vaccine but indicated they were unsure if they would or not (hesitant) were asked what would help them to make a decision (*n* = 490).

The survey enabled respondents who indicated that they would not receive the vaccine to be asked additional questions in an effort to determine why they made that decision. For this question, the respondents could select as many choices as applied to them. The two most commonly cited reasons were concerns with the speed and thoroughness of testing of the vaccines, and the fear of side effects (Figure 1). The next most common reason was a lack of trust in the people advocating for the vaccines, followed by general anti-vaccine attitudes. 

Since the question asking about intent to be vaccinated had an option for “unsure,” those who were unsure (hesitant) were further queried about what would help them make a decision about vaccination. Here, the answers were also grouped, with the most common responses indicating that more time or testing on a larger number of people would be most effective. Of the responses where action could make a difference, having the vaccine receive full FDA approval was the most commonly selected option (30.81%), followed by having a conversation with a doctor (19.59%). Since this study was comprised of a population where the vaccine was available and in fact encouraged their employment, vaccine access was not a major issue, with only 0.4% indicating that a “closer location” and only 1.6% saying “increased access” would help them decide to be vaccinated (Figure 1).

### 3.6. Machine Learning Analysis

In order to determine which answers were most associated with a respondent being COVID-19 “vaccine hesitant” we applied a random forest machine learning algorithm, which generates a computational model to predict classification of data based on a discreet variable (e.g., vaccine hesitancy) and other variables [17]. This approach was specifically selected since so many different factors/variables were significantly associated using more traditional statistical methods. Our approach used the mean decrease in Gini value, a quantitative metric of uncertainty, for each question or feature. Ranking the list of factors/features by this value enabled us to construct a list of the best predictors for vaccine hesitancy (Table 5). All other questions had Gini values that were extremely low. Overall, the model was able to accurately classify people answering “Unsure” about whether they would be vaccinated 100% of the time, while the model was unable to accurately classify (0%) those answering “No” when asked if they would be vaccinated. Overall, the accuracy rate of this method was 64.8% and a receiver–operator characteristic curve showed an overall performance of 65.2%. This suggests that those answering “No” had no consistent associated answers, while the survey identified several factors/features that associate with those answering “Unsure” (Table 5). 

Supporting the demographic analysis above, the machine learning approach identified work role, age, and education as being highly important in determining whether a respondent would be vaccine-hesitant. Attitudes about the severity of COVID-19 and vaccinations in general were also highly associated with this role, with “How has your experience with COVID-19 changed your overall opinion on vaccinations” the top predictors and “Based on your overall experience, how serious is COVID-19” at number 5. Interestingly, the location where a respondent works was highly associated with COVID-19 vaccine hesitancy.

### 3.7. Location of Respondents

New York City is diverse, with different neighborhoods having different characteristics. The hospitals in the system were differentially affected by the pandemic, with the facilities in Queens and Bronx bearing the brunt of cases. With this background, we aimed to determine if the location of work is a significant factor in terms of vaccine hesitancy (Table 5). 

We found that the vaccination status of respondents was dependent on the borough in which they worked (*p* < 0.001). Queens was the borough with the highest level of vaccination, at 84%, while Staten Island was by far the lowest, at 62%. However, the number of respondents who worked in Staten Island was considerably lower than the other boroughs (only 45 responses), so this finding may be biased. Intent to be vaccinated significantly (*p* < 0.01) depended on work location. Queens had the lowest proportion of hesitant respondents, at 11%, and had a low proportion of refusers, at only 4%. Staten Island had a high proportion of vaccine hesitant respondents (26%) and refusers (13%).

We evaluated whether where the respondents lived had an effect on vaccination status, and found a significant association (*p* < 0.001). In this case, those living in Westchester were most likely to be vaccinated, at 88%, and those living in the Bronx were least likely, at 70%. Among the unvaccinated, those with the highest ratio of hesitant to refusers lived Upstate (4.1:1), whereas in Staten Island, the number of vaccine refusers was nearly as high as hesitant individuals (ratio of 1.22:1).

Since the impact of COVID-19 was different between boroughs, we used ANOVA to determine whether there was any significant difference in how the seriousness of the pandemic was perceived between boroughs. We observed no significant overall difference in how respondents rated the seriousness of COVID-19 based on where they live (*p* = 0.17). 

**Figure 2:**
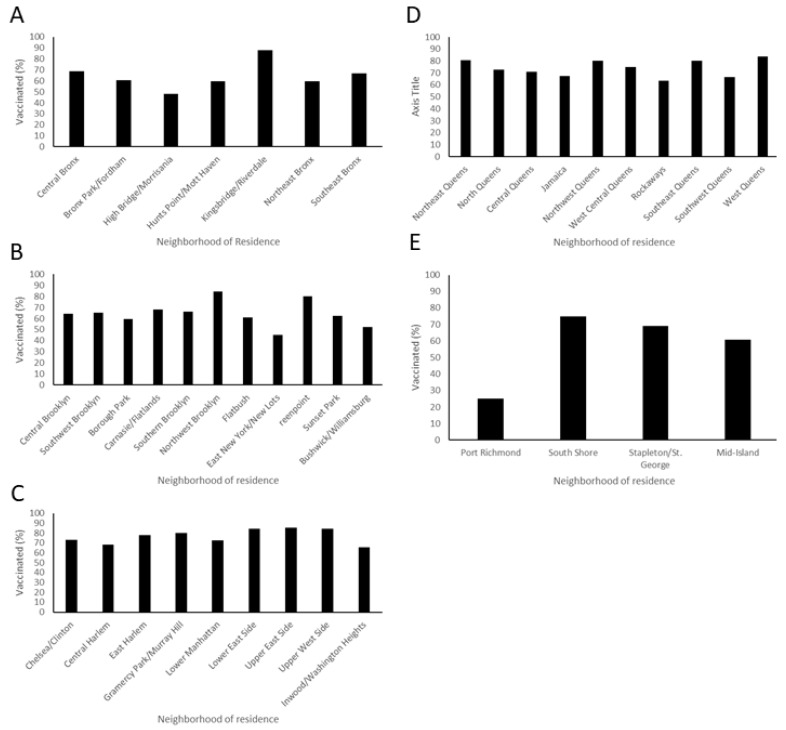
**Neighborhoods within boroughs show different vaccination rates between health professionals who live there**. (**A**) Vaccination rates in the Bronx are significantly different based on neighborhood (*p* < 0.001, *n* = 479) and range from 87.75% to 48.33%. (**B**) Vaccination rates for health professionals in Brooklyn are dependent on neighborhood (*p* = 0.00028 *n* = 762). Vaccination rates in Brooklyn range from 45.24% to 84.62%. (**C**) Manhattan has overall high vaccination rates but they are significantly dependent on neighborhoods (*p* = 0.0169 *n* = 593). Health professionals vaccinated in Manhattan range from 65.57% to 85.59%. (**D**) Queens was more homogenous in HCW vaccination rate by neighborhood, with no overall significant difference found (*n* = 624). Rates in Queens range from 63.33% to 83.63%. (**E**) Staten Island vaccination rates significantly differed by neighborhood (*p* = 0.022, *n* = 89).

Within each borough, there are many neighborhoods with substantial differences in population attributes, including vaccination rates. We used ZIP codes to determine the specific neighborhoods where the respondents lived within each borough, and these were compared for differences in vaccination rates. HCW in the Bronx demonstrated a dependence on vaccination rate by neighborhood (*p* < 0.0021). The most vaccinated location in this borough was Kingsbridge, with 87.75% vaccinated, and the lowest was High Bridge, with 48.33% vaccinated (Figure 2).

Similarly, Brooklyn neighborhoods were significantly different in vaccination rates of the health care professionals who lived there (*p* = 0.00028). Brooklyn neighborhoods seemed to cluster into three groups. Greenpoint and Northwest Brooklyn had high levels of vaccination (over 80%). East New York/New Lots and Bushwick/Williamsburg had low levels, with less than 60% vaccinated. The rest of the neighborhood health care workers had between 60 and 80% rates (Figure 2).

Manhattan, interestingly, showed a difference by neighborhood (*p* = 0.017) but the variance between neighborhoods was less pronounced. No neighborhood in Manhattan had hospital workers vaccinated at less than 65%. The neighborhood with the lowest rate was Washington Heights, with 66% vaccinated and that with the highest was the Upper East Side, with 85.59%.

Queens did not demonstrate a significant difference between neighborhoods. Staten Island did show a significant difference between neighborhoods (*p* = 0.022). Staten Island also had the neighborhood with the lowest vaccination rate, Port Richmond, at only 25%. However, this result is based on only 12 respondents in that neighborhood.

## 4. Discussion

Vaccine hesitancy is a significant and complex challenge to public health. It is a spectrum including attitudes and behaviors ranging from complete refusal to complete acceptance of vaccination [18,19]. It has led to a delay in getting vaccinated or modification of schedule of vaccination (partial vaccination) [20]. Historically, vaccine hesitancy has been impacted by cultural, social, and political influences in addition to vaccine related factors [21,22].

We performed a survey-based study to understand the factors affecting COVID-19 vaccine attitudes among healthcare professionals in New York City, at a time when the vaccine was new. The results of this study show that a large variety of factors are associated with vaccine attitudes between HCW. These include demographic factors, education, work role, attitudes about the pandemic, and the location of where the respondent lives and works. Notably, we observed that in the early period of the vaccine rollout, the rates of vaccine hesitancy/refusal among HCW were comparable to the general population in New York City [23]. This observation was remarkable, as HCW have been on the frontlines caring for the COVID-19 patients and are trusted sources of vaccine information, with an ability to influence the “choice to vaccinate” for their patients and social contacts [24].

### 4.1. Demographics and COVID-19 Vaccine Hesitancy

Demographic factors, including age, race, and gender, were all significantly associated with hesitancy toward the COVID-19 vaccine. The Household Pulse Survey also demonstrated that COVID-19 vaccine hesitancy was higher among women when compared to men, younger age groups when compared to respondents over 65 years and participants with college degrees compared to those without a degree [25]. Our results are comparable to the data from the Kaiser Family Foundation/The Undefeated survey, which demonstrated that among HCWs there is a substantial cohort that is vaccine hesitant, especially among Black HCW [26]. The role at the hospital was confirmed to be an important factor in COVID-19 vaccine hesitancy by both the χ2 analysis and the machine learning analysis. Notably, we observed a significantly lower vaccine acceptance among maintenance/environmental staff, hospital police and pharmacies, while Medical staff including physicians, residents, nurse practitioners, and physician assistants were highest. Remarkably, those who were directly addressing COVID-19 (community outreach tracers) had some of the lowest levels of vaccination and the highest hesitancy. It was notable that nurses, who have the greatest contact with patients, had a high rate of vaccine hesitancy in spite of being a trusted source of medical information for the patient. A cross-sectional study of nurses in Hong Kong showed that less than two-thirds intended to get the COVID-19 vaccine when available [27]. We believe that a significant reason for HCW in the child-bearing age group being hesitant to COVID-19 vaccination in our survey is because the survey was administered in the initial phase of the vaccine roll out, when there was a paucity of comparative data on vaccine studies in pregnant women and its correlation with birth outcomes [28,29]. Social scientists have commented that people who reject vaccines harbor a transformation of their core beliefs about what they owe one another and are not scientifically less literate or knowledgeable than those who accept vaccination [30]. During the 1950 polio campaign, Americans accepted vaccination as a civic duty. However, in the following years, the “choice” of an individual’s health became their personal responsibility, in spite of a multitude of factors, especially “social determinants of health” that are beyond the individual’s control and impact their choices. These have contributed to a shift in people’s mind, from the idea of the common good. 

### 4.2. Location of Work and Home

The geographic pattern of vaccine hesitancy is also significant. Nationally, it has been observed that hesitancy rates are lowest on the North East and West coast and highest in the South, Mid-West/Central US and Alaska with higher rates of hesitancy in rural communities when compared to urban ones [25,31]. The COVID-19 pandemic has disproportionately affected communities of color in NYC. Neighborhood social disadvantage contributed to higher number of infections in these communities [32]. The lowest vaccination rates by borough, as per NYC Department of Health and Mental Hygeine data 31 May 2021 were Flatlands/Midwood, Brooklyn (33% received at least one dose), Edgemere/Far Rockaway, Queens (31% received at least one dose), Central Harlem/Washington heights in Upper Manhattan (37% received at least one dose), Hunts Point, Bronx (34% received at least one dose) with Tottenville, Staten Island (39% had at least one dose). Where a HCW lived and worked was associated with their vaccination status and hesitant attitudes. These varied by both borough and by neighborhood within boroughs. Infection rates, death, and hospitalization rates of specific boroughs did not correspond to these vaccine attitudes in the boroughs. For example, Staten Island had significantly more infections per 100,000 than any other borough (*p* < 0.0001, test of proportions) [33], yet Staten Island-based medical personnel had the lowest rates of vaccination and pro-vaccine attitudes.

### 4.3. First-Hand Experience with Serious COVID-19 Cases

The best predictor of vaccine hesitancy according to the random forest analysis was how the COVID-19 pandemic had affected the respondents’ views towards vaccines in general, a finding that is concerning, as it suggests that people who are hesitant towards the COVID-19 vaccines may transfer that hesitancy to other vaccines, exacerbating the already significant vaccine hesitancy problem. Another attitude that was predictive of vaccine hesitancy was how important the respondent felt the pandemic was. Truthful information and experience with people who have suffered difficult consequences of the pandemic may be helpful in promoting vaccine acceptance among these people. 

When asked why they had chosen to not receive a vaccine, “It was developed too quickly”, and “I don’t want to be experimented on” were the most prevalent answers. In other studies, we have found that attitudes towards vaccines in general are highly influential in making vaccine decisions [2,34], and it is likely that those with a general level of distrust in vaccines will indicate greater concern about the speed of development or the lack of testing. 

### 4.4. Changing Attitudes over Time

Multiple studies have demonstrated a reduction in vaccine hesitancy over time [21,31]. The greatest declines in vaccine hesitancy were observed among participants in the 18–24-year age group, women and non- Hispanic Black persons as per the Household Pulse Survey results. Additionally, participants with a lower income, a younger age, and without insurance or on Medicaid were more likely to remain vaccine hesitant over time. The higher vaccine hesitancy rates among Black respondents improved over time when compared to rates among the white population. Unfortunately, the percentage of vaccine refusers have remained constant in spite of time in multiple studies [35,36]. Hence, interventions to improve vaccination uptake are likely to succeed if focused on the vaccine hesitant group rather than on the vaccine refusers [21]. 

### 4.5. Strategies for Intervention

Targeted messaging directed toward the hesitant population delivered by their trusted individuals will be effective in changing minds. Engaging vaccinated individuals from hesitant/minority communities, and developing communication aids that are culturally, linguistically, and literacy appropriate will help promote vaccination in vulnerable communities [37]. The neighborhood-level differences in vaccination (Figure 2) support the need for highly targeted interventions to improve vaccine hesitancy. Multiple health behavior models, including threat and coping appraisal, cues to action, self-efficacy, perceived benefits/barriers, perceived behavior control, and attitudes and contextual factors are known to influence routine vaccination. Applications of these models to fight the COVID-19 pandemic would be a major tool to combat vaccine hesitancy [38]. The geographical variance in vaccine acceptance suggests that while exposure to the disease is important, attitudes are moderated by other region-specific factors. Social attitudes that have not been captured by the survey may be highly important in determining attitudes towards infection severity and vaccination. Perhaps vaccination messaging that addresses the unique features of these locations would be effective in promoting COVID-19 vaccination or similar vaccination campaigns in the future.

Among the determinants of vaccine hesitancy, communication and media environment are of paramount importance. Negative communications have long term effects on public perception. For example, there is controversy over the association of the measles vaccine and autism, hepatitis B vaccine and Multiple Sclerosis [39]. News about serious adverse events (thrombosis) linked to the Janssen COVID-19 vaccine reduced confidence among the unvaccinated/hesitant especially the Hispanic women [36]. 

“Unspoken vaccine hesitancy” is a state when HCWs do not voice their concerns about vaccination among their peers or in the workplace due to institutional and societal pressures to vaccinate, especially in the setting of tension and polarization across communities about vaccination [6]. Additionally, studies have demonstrated that HCWs experience significant anxiety, depression and psychosocial distress related to working in the COVID-19 era [40]. Addressing vaccine hesitancy in these situations is difficult. Innovative multidisciplinary approaches to openly discuss vaccine-related concerns among these hesitant HCWs will build confidence and go a long way towards resolving their fear and anxiety.

Vaccine mandates in healthcare institutions are an attempt to protect the patients based on the ethical principle to do no harm and to create a safe environment for them. Health + Hospitals enforced the state-recommended vaccine mandate to have all HCW receive at least one dose of the COVID-19 vaccine by 27 September 2021. This initiative reduced vaccine hesitancy further and forced the exit of vaccine refusers from the workforce. There have been multiple interventions internationally, like green pass implementation, domestic vaccine passports etc. [12], to improve vaccination uptake. In the background of waning immunity following COVID-19 [41,42] vaccination and the need to boost doses to combat arising new SARS-CoV-2 variants of concern, there remains a continued role for mandates due to persistent vaccine hesitancy. While vaccine mandates affect an individual’s choice (right to refuse vaccination), it is critical to remember that every HCW has an ethical and moral responsibility to do no harm and to place their patient’s health goals and interests above their own [43].

### 4.6. Limitations

This survey was performed early in the vaccine rollout process. Our other work from approximately the same time indicates that the political influence on vaccine acceptance in the United States was not a major factor at that time [34], although it certainly is now [44]. This study also does not consider more forceful measures, such as mandates, that have increased the vaccination percentages in New York City, as well as in other locations [45]. We hope that this work will indicate the directions and areas of focus for vaccine messaging and communication in the future, as well as obstacles and issues that need to be overcome if a similar situation were to arise again. 

## Figures and Tables

**Table 1:** Demographic characteristics of respondents.

Data	*n*	%
**Age (*n* = 3493)**		
18–24	77	2.2%
25–34	692	19.8%
35–44	746	21.4%
45–54	817	23.4%
55–54	880	25.2%
65+	281	8.0%
**Gender (*n* = 3491)**		
Female	2491	71.4%
Male	918	25.3%
Non-binary/third gender	19	0.5%
Prefer not to answer	63	1.8%
**Ethnicity (*n* = 3474)**		
Hispanic or Latino/a/x	698	20.1%
Non-Hispanic	2776	79.9%
**Race (*n* = 3632)**		
American Indian	61	1.8%
Asian	680	18.7%
Black or African	942	25.9%
Hispanic, Latino	257	7.1%
Pacific Islander or Native Hawaiian	31	0.9%
White	1492	41.0%
Mixed Race	125	3.4%
Other	19	0.5%
Prefer not to answer	27	0.7%
**Number of Children (*n* = 3421)**		
0	1327	38.7%
1	621	18.1%
2	899	26.2%
More then 2	584	17.0%
**Education (*n* = 3465)**		
Some secondary school	6	0.2%
GED	38	1.1%
High school diploma	119	3.4%
Some college	289	8.3%
Associate’s degree	284	8.2%
Bachelor’s degree	903	26.1%
Some graduate school	137	4.0%
Master’s degree	862	24.9%
Doctoral level	820	23.7%
Other	7	0.2%

**Table 2:** Primary role in the health care system.

Primary Role (*n* = 3287)	*n*	%
Physician/Attending	562	17.1%
Administrative Support Staff	507	15.4%
Central Office Administration	222	6.8%
Hospital Police	162	4.9%
Patient Care Associate	160	4.9%
Nurse Practitioner	91	2.8%
Nurse	580	17.6%
Resident	79	2.4%
Physician Assistant	76	2.3%
Social Worker	59	1.8%
Information Technology	46	1.4%
Maintenance Staff	25	0.8%
Environmental services	25	0.8%
Hospital Administration	23	0.7%
Medical Student	20	0.6%
Dietary Services/Nutritionist	10	0.3%
Other	640	19.5%

**Table 3:** Associations between demographics and COVID-19 vaccine status.

Demographic Variable	Vaccinated	Hesitant	*p* Value	Refuser	*p* Value	Hesitant Effect Size (IQ)
**Age group**			<0.001		<0.001	
**18–24**	34 (1%)	14 (3%)		12 (5%)		2.91
**25–34**	471 (18%)	99 (20%)		51 (22%)		0.36
**35–44**	509 (19%)	101 (21%)		78 (34%)		0.36
**45–54**	592 (22%)	107 (22%)		42 (83%)		−0.36
**55–64**	664 (25%)	103 (21%)		23 (10%)		−0.73
**>65**	235 (9%)	19 (4%)		5 (2%)		−2.91
**Race**			<0.001		<0.001	
**Native American**	19 (0.8%)	7 (1.6%)		3 (1%)		0.45
**Asian**	547 (22%)	40 (9.2%)		7 (3%)		−1.25
**Black**	486 (20%)	188 (43%)		103 (49%)		0.45
**Hispanic**	174 (7%)	34(8%)		22 (11%)		−0.45
**Native Hawaiian**	17 (1%)	5 (1%)		2 (1%)		0.15
**White**	1167 (47%)	145 (33%)		60 (29%)		−0.85
**Mixed**	47 (2%)	11 (2%)		4 (2%)		−0.15
**Other**	10 (0.4%)	4 (1%)		2 (1%)		0.55
**Gender**			<0.001		<0.001	
**Female**	1723 (64%)	348 (71%)		166 (72%)		−0.35
**Male**	735 (27%)	73 (15%)		36 (16%)		−0.90
**Non binary**	10 (0.4%)	6 (1%)		2 (1%)		0.98
**prefer not to answer**	36 (1%)	15 (3%)		10 (4%)		0.35

Hesitant effect size measures proportion of interquartile range from the median for each item. Negative numbers are less likely to be vaccine-hesitant.

**Table 4:** Association between education and hospital role with COVID-19 vaccine status.

	Vaccinated	Hesitant	*p* Value	Refused	*p* Value	Hesitant Effect Size
**Education**			**<0.001**		**<0.001**	
Some high school	5 (0.2%)	1 (0.2%)		0		−0.17
GED	19 (1%)	10 (2%)		5 (2.2%)		1.04
High school diploma	70 (3%)	24 (5%)		7 (3%)		1.04
Some college	174 (6%)	49 (10%)		36 (16%)		0.17
Associates degree	177 (7%)	49 (10%)		27 (12%)		0.17
Bachelor’s degree	600 (22%)	137 (28%)		67 (29%)		−0.17
Some graduate school	91 (3%)	20 (4%)		9 (4%)		−0.17
Master’s degree	605 (23%)	113 (23%)		41 (18%)		−0.52
Doctoral degree	744 (28%)	30 (6%)		14 (6%)		−2.43
Other	3 (0.1%)	2 (0.4%)		2.9 (1%)		1.22
**Primary Role**	**Vaccinated**	**Hesitant**	***p* < 0.001**	**Refused**	***p* < 0.001**	
Medical staff	820 (31%)	30 (6%)		16 (7%)		−0.76
Nursing and support staff	502 (19%)	110 (22%)		56 (24%)		0
Maintenance/Environmental staff	32 (1%)	6 (1%)		5 (2.2%)		−0.12
Hospital Administrative staff	662 (25%)	203 (41%)		83 (36%)		0.29
Clinical support staff	348 (13%)	36 (7%)		23 (10%)		−0.41
Hosp Police	9 (0.3%)	6 (1%)		8 (3%)		0.59
Community Outreach tracers	38 (1%)	19 (4%)		2 (1%)		0.94
Pharmacy	38 (1%)	4 (1%)		3 (1.3%)		−0.41
Other	0	2 (1%)		2 (1%)		2

Hesitant effect size measures proportion of interquartile range from the median for each item. Negative numbers are less likely to be vaccine hesitant.

**Table 5:** Machine learning analysis of survey questions associated with vaccine hesitancy.

Question	Mean Decrease Gini
How has your experience with COVID-19 changed your overall opinion on vaccinations?	8.2
Which best describes your primary role at work?	7.97
What is your age group?	5.86
What is your highest level of formal education?	4.58
Based on your overall experience how serious is COVID-19?	2.72
Where do you work?	2.35
How many children do you have?	2.16
What is your gender?	1.41
I worry that I cannot pay for the vaccine now or in the future	1.16
Are you Hispanic or Latino?	0.71

## Data Availability

Data are available upon request to the corresponding authors.

## Supplementary Materials

The following supporting information can be downloaded at: https://www.mdpi.com/article/10.3390/vaccines10020273/s1, Table S1: complete survey.
