# Supplementary material for: Impact of Local and Demographic Factors on Early COVID-19 Vaccine Hesitancy among Health Care Workers in New York City Public Hospitals
Source: Vaccines (Basel). 2022 Feb 10;10(2):273. doi: 10.3390/vaccines10020273 (PMC8879070; doi:10.3390/vaccines10020273)
Supplement: Supplementary file 1 [file vaccines-10-00273-s001.zip › vaccines-1576248-supplementary.pdf]

NYC HEALTH+ HOSPITALS

# Knowledge, Attitude, and Perceptions of COVID-19 Vaccination among Healthcare Workers in New York City

## COVID-19 Vaccine Survey

**Thank you for taking this survey. Your responses will help us understand how you feel about the COVID-19 vaccine. Your answers will be anonymous; we will not know who answered these questions.**

1. Did you get the COVID-19 vaccine?

- ◯ Yes
- ◯ No

Yes->Q2
No->Q6

NYC HEALTH+ HOSPITALS

## Knowledge, Attitude, and Perceptions of COVID-19 Vaccination among Healthcare Workers in New York City

2. Why did you get the vaccine? Select one or more answers.

- [ ] I am at risk for getting COVID-19 because of my age and/or other medical issues
- [ ] I work on the frontline
- [ ] I live with or take care of someone who is at risk (a person who is 65 years or older and/or who has medical issues that make them more likely to become sick)
- [ ] I don't want to become sick with COVID-19
- [ ] My doctor (or person who provides medical care) suggested getting the vaccine
- [ ] My employer recommended getting the vaccine
- [ ] I heard on the news or social media that it is recommended
- [ ] I want to help control the spread of COVID-19
- [ ] Other (please specify)

NYC HEALTH+ HOSPITALS

## Knowledge, Attitude, and Perceptions of COVID-19 Vaccination among Healthcare Workers in New York City

3. Did you experience any of the following after getting the vaccine? Select one or more answers.

- [ ] Arm pain or soreness
- [ ] Felt tired or exhausted
- [ ] Fever
- [ ] Body aches
- [ ] Rash or any allergy
- [ ] I don't know
- [ ] Other (please specify)

  ____________________

- [ ] None of the above

NYC HEALTH+ HOSPITALS

## Knowledge, Attitude, and Perceptions of COVID-19 Vaccination among Healthcare Workers in New York City

4. Will you get your second shot as scheduled?

- Yes
- No
- I don't know

Yes->Q9
No->Q5
I don’t know->Q5

NYC HEALTH+ HOSPITALS

## Knowledge, Attitude, and Perceptions of COVID-19 Vaccination among Healthcare Workers in New York City

5. Why will you not (or possibly not) get your second shot? Select one or more answers.

- [ ] One shot is enough
- [ ] I had a bad reaction after the first shot
- [ ] I heard side effects after the second shot can be bad
- [ ] Busy schedule
- [ ] Other (please specify)

All Choices -->Q9

NYC HEALTH+ HOSPITALS



6. I will get the vaccine within the next two months.

- Agree
- Disagree
- I don't know

Agree->Q9
Disagree->Next Question
I don’t know->Q8

NYC HEALTH+ HOSPITALS

## Knowledge, Attitude, and Perceptions of COVID-19 Vaccination among Healthcare Workers in New York City

7. Why will you not get the vaccine in the next two months? Select one or more answers.

All responses-->Q9

- [ ] I do not believe in vaccines
- [ ] The COVID-19 vaccine does not work
- [ ] I could get COVID-19 from the vaccine
- [ ] I do not work on the frontline
- [ ] I do not need/want another shot
- [ ] I do not want a vaccine made outside of the United States
- [ ] It was developed too quickly
- [ ] My faith does not allow me to get the vaccine
- [ ] The sides effects can be bad
- [ ] The vaccine was not tested on people like me or with my condition
- [ ] I don't want to be experimented on
- [ ] My doctor (or person who provides medical care) said I should not get it
- [ ] I do not trust the people who suggested I get the vaccine
- [ ] I am pregnant or breastfeeding (or will become pregnant or breastfeed in the near future)
- [ ] I am not eligible to get the vaccine
- [ ] Other (please specify)

NYC HEALTH+ HOSPITALS

## Knowledge, Attitude, and Perceptions of COVID-19 Vaccination among Healthcare Workers in New York City

8. What would help you make a decision about getting the vaccine? Select one or more answers.

- [ ] I need more time to decide
- [ ] I need more information
- [ ] I want to talk to my doctor (or person who provides medical care)
- [ ] I want to talk to someone I trust (community leader, religious leader, etc)
- [ ] I will decide after I am no longer pregnant or breastfeeding
- [ ] I will decide after the vaccine has full approval from the FDA (Food and Drug Administration)
- [ ] I want to wait until more people get the vaccine
- [ ] I don't know
- [ ] Other (please specify)

NYC HEALTH + HOSPITALS

## Knowledge, Attitude, and Perceptions of COVID-19 Vaccination among Healthcare Workers in New York City

9. How has your experience with COVID-19 changed your overall opinion on vaccinations?

- ◯ I am much more likely to vaccinate myself/my children
- ◯ I am more likely to vaccinate myself/my children
- ◯ My opinion on vaccinations has not changed
- ◯ I am less likely to vaccinate myself/my children
- ◯ I am much less likely to vaccinate myself/my children

## Knowledge, Attitude, and Perceptions of COVID-19 Vaccination among Healthcare Workers in New York City

10. I worry that I cannot pay for the vaccine (now or in the future).

- ◯ Agree
- ◯ Disagree
- ◯ I don't know

NYC HEALTH+ HOSPITALS

## Knowledge, Attitude, and Perceptions of COVID-19 Vaccination among Healthcare Workers in New York City

11. What do you think would happen if you became sick with COVID-19? Select one or more answers.

- [ ] I would likely have no symptoms or a mild disease
- [ ] I could become ill but not need hospitalization
- [ ] I could be hospitalized
- [ ] I could become very ill and need intensive care
- [ ] I could die
- [ ] I don't know

Knowledge, Attitude, and Perceptions of COVID-19 Vaccination among Healthcare Workers in New York City

12. Based on your overall experience, how serious is COVID-19?

- ◯ Not a problem at all
- ◯ Not as bad as other problems in the United States
- ◯ Somewhat of a problem
- ◯ A severe problem, more important than most issues
- ◯ The most important problem in the United States

NYC HEALTH+ HOSPITALS

## Knowledge, Attitude, and Perceptions of COVID-19 Vaccination among Healthcare Workers in New York City

13. Where do you get most of your information on COVID-19? Select one or more answers.

Work/ NYC HHC-->Q14
All other responses -->Q15

- [ ] Work/ NYC Health + Hospitals
- [ ] My doctor (or person who provides medical care)
- [ ] Other medical or scientific professionals
- [ ] Public health organizations like the CDC (Center for Disease Control and Prevention), or NYC DOH (New York City Department of Health)
- [ ] Communications from professional organizations
- [ ] Public/political figures
- [ ] Scientific research or review studies
- [ ] Friends or family
- [ ] My religious community
- [ ] News organizations (print, TV, or online)
- [ ] Social media (Facebook, Twitter, TikTok)
- [ ] None of the above

Other (please specify)

Knowledge, Attitude, and Perceptions of COVID-19 Vaccination among Healthcare Workers in New York City

14. You selected that you get COVID-19 information from Health + Hospitals. Which resource do you find most helpful?

- [ ] COVID-19 Resource Hub
- [ ] Staff emails
- [ ] Staff meetings
- [ ] Insider articles you read on the intranet
- [ ] Flyers, brochures, and posters with information you see and receive
- [ ] My supervisor or coworkers
- [ ] Other (please specify)

Knowledge, Attitude, and Perceptions of COVID-19 Vaccination among Healthcare Workers in New York City

15. What is your age group ?

- ◯ 18-24
- ◯ 25-34
- ◯ 35-44
- ◯ 45-54
- ◯ 55-64
- ◯ 65+

NYC HEALTH+ HOSPITALS

Knowledge, Attitude, and Perceptions of COVID-19 Vaccination among Healthcare Workers in New York City

16. What is your gender?

- ◯ Female
- ◯ Male
- ◯ Non-binary/third gender
- ◯ Prefer not to answer

Prefer to self-describe

NYC HEALTH + HOSPITALS

## Knowledge, Attitude, and Perceptions of COVID-19 Vaccination among Healthcare Workers in New York City

17. Are you Hispanic or Latino/a/x?

- ◯ Yes
- ◯ No

NYC HEALTH+ HOSPITALS

Knowledge, Attitude, and Perceptions of COVID-19 Vaccination among Healthcare Workers in New York City

18. Regardless of your answer to the prior question, please indicate how you identify yourself (you may select one or more).

- [ ] American Indian or Alaska Native
- [ ] Asian
- [ ] Black or African American
- [ ] Native Hawaiian or other Pacific Islander
- [ ] White
- [ ] Other (please specify)

NYC HEALTH + HOSPITALS

Knowledge, Attitude, and Perceptions of COVID-19 Vaccination among Healthcare Workers in New York City

19. Please enter the zip code where you live.

NYC HEALTH+ HOSPITALS

## Knowledge, Attitude, and Perceptions of COVID-19 Vaccination among Healthcare Workers in New York City

20. What is your highest level of formal education?

- ◯ Some secondary school/high school
- ◯ GED
- ◯ High school diploma
- ◯ Some college
- ◯ Associate's degree
- ◯ Bachelor’s degree
- ◯ Some graduate school
- ◯ Master’s degree
- ◯ Doctoral level
- ◯ Other (please specify)

NYC HEALTH+ HOSPITALS

## Knowledge, Attitude, and Perceptions of COVID-19 Vaccination among Healthcare Workers in New York City

21. How many children do you have ?

- ○ 0
- ○ 1
- ○ 2
- ○ More than 2

## Knowledge, Attitude, and Perceptions of COVID-19 Vaccination among Healthcare Workers in New York City

22. Which best describes your primary role at work?

- ◯ Physician/Attending
- ◯ Nurse Practitioner
- ◯ Physician Assistant
- ◯ Nurse
- ◯ Resident
- ◯ Medical Student
- ◯ Patient Care Associate
- ◯ Maintenance Staff
- ◯ Environmental Services
- ◯ Administrative Support Staff
- ◯ Physiotherapy, Occupational Therapy, Speech and Swallow
- ◯ Social Worker
- ◯ Hospital Police
- ◯ Dietary Services/Nutritionist
- ◯ Hospital Administration
- ◯ Information Technology
- ◯ Central Office Administration /Operational Offices

Other (please specify)

NYC HEALTH+ HOSPITALS

## Knowledge, Attitude, and Perceptions of COVID-19 Vaccination among Healthcare Workers in New York City

23. Where do you work?

- The Bronx
- Brooklyn
- Manhattan
- Queens
- Staten Island

NYC HEALTH+ HOSPITALS

## Knowledge, Attitude, and Perceptions of COVID-19 Vaccination among Healthcare Workers in New York City

You have reached the end of the survey.  Thank you very much for participating!
